# Dendritic Cells from Oral Cavity Induce Foxp3^+^ Regulatory T Cells upon Antigen Stimulation

**DOI:** 10.1371/journal.pone.0051665

**Published:** 2012-12-18

**Authors:** Sayuri Yamazaki, Akira Maruyama, Kohei Okada, Misako Matsumoto, Akimichi Morita, Tsukasa Seya

**Affiliations:** 1 Department of Microbiology and Immunology, Graduate School of Medicine, Hokkaido University, Sapporo, Japan; 2 Department of Geriatric and Environmental Dermatology, Nagoya City University, Graduate School of Medical Sciences, Nagoya, Japan; The Ohio State University, United States of America

## Abstract

Evidence is accumulating that dendritic cells (DCs) from the intestines have the capacity to induce Foxp3^+^CD4^+^ regulatory T cells (T-regs) and regulate immunity versus tolerance in the intestines. However, the contribution of DCs to controlling immunity versus tolerance in the oral cavity has not been addressed. Here, we report that DCs from the oral cavity induce Foxp3^+^ T-regs as well as DCs from intestine. We found that oral-cavity-draining cervical lymph nodes contained higher frequencies of Foxp3^+^ T-regs and ROR-γt^+^ CD4^+^T cells than other lymph nodes. The high frequency of Foxp3^+^ T-regs in the oral-cavity-draining cervical lymph nodes was not dependent on the Toll like receptor (TLR) adaptor molecules, Myd88 and TICAM-1 (TRIF). In contrast, the high frequency of ROR-γt^+^ CD4^+^T cells relies on Myd88 and TICAM-1. *In vitro* data showed that CD11c^+^ DCs from oral-cavity-draining cervical lymph nodes have the capacity to induce Foxp3^+^ T-regs in the presence of antigen. These data suggest that, as well as in the intestinal environment, antigen-presenting DCs may play a vital role in maintaining tolerance by inducing Foxp3^+^ T-regs in the oral cavity.

## Introduction

Foxp3^+^CD25^+^CD4^+^ regulatory T cells (T-regs), constitute about 5–10% of peripheral CD4^+^T cells and control immunological self-tolerance in rodents and human [1], [2], [3], [4]. The expansion and induction of CD25^+^Foxp3^+^ T-regs in the periphery are controlled by professional antigen-presenting cells, dendritic cells (DCs) [5], [6]. DCs can expand thymic-derived natural occurring T-regs [7], [8], [9]. DCs are the most efficient antigen presenting cells to induce Foxp3^+^T-regs from Foxp3^−^ precursors in the periphery [10], [11]. Peripheral DCs directly control the numbers and homeostasis of Foxp3^+^T-regs *in vivo* [12].

Foxp3^+^T-regs induced by DCs in the intestine control the balance between inflammation and tolerance in the gut [13], [14], [15]. CD103^+^DCs in the intestine use the retinoic acid-metabolizing enzyme retinaldehyde dehydrogenase and induce Foxp3^+^T-regs to maintain oral tolerance [16], [17]. Intestinal DCs use signaling through ß-catenin to induce Foxp3^+^T-regs, which suppress Th17 and Th1 responses in the intestine [18]. Specific pathogens or Toll-like receptor (TLR) signals have been shown to induce Foxp3^+^T-regs in the intestine [19], [20]. Moreover, Foxp3^+^T-regs control Th17 cells using interleukin (IL)-10 in the intestine [21], [22]. Thus, Foxp3^+^T-regs in the intestine are important in maintaining mucosal tolerance where there are vast numbers of commensal microbes and food antigens.

As in the intestine, many commensal microbes and food antigens also exist in the oral cavity [23], [24], [25], [26]. Oral cavity is often involved with systemic immunological diseases such as graft versus host diseases, Stevens-Johnson syndrome, Behçet diseases, pemphigus vulgaris and Sjögren’s syndrome. In addition, oral cavity is the place where many viruses, including influenza, herpes, common cold etc., start to infect. Therefore, it is important to identify how immune response is regulated in the oral cavity. Here we found that the DCs from oral cavity have the capacity to induce Foxp3^+^T-regs. To our knowledge, this is the first report showing that DCs from the oral cavity induce Foxp3^+^T-regs to maintain tolerance.

## Results

### The Frequency of Foxp3^+^T-regs is Increased in Cervical Lymph Nodes (CLNs) in a Myd88/TICAM-1- Independent Manner

**Figure 1:**
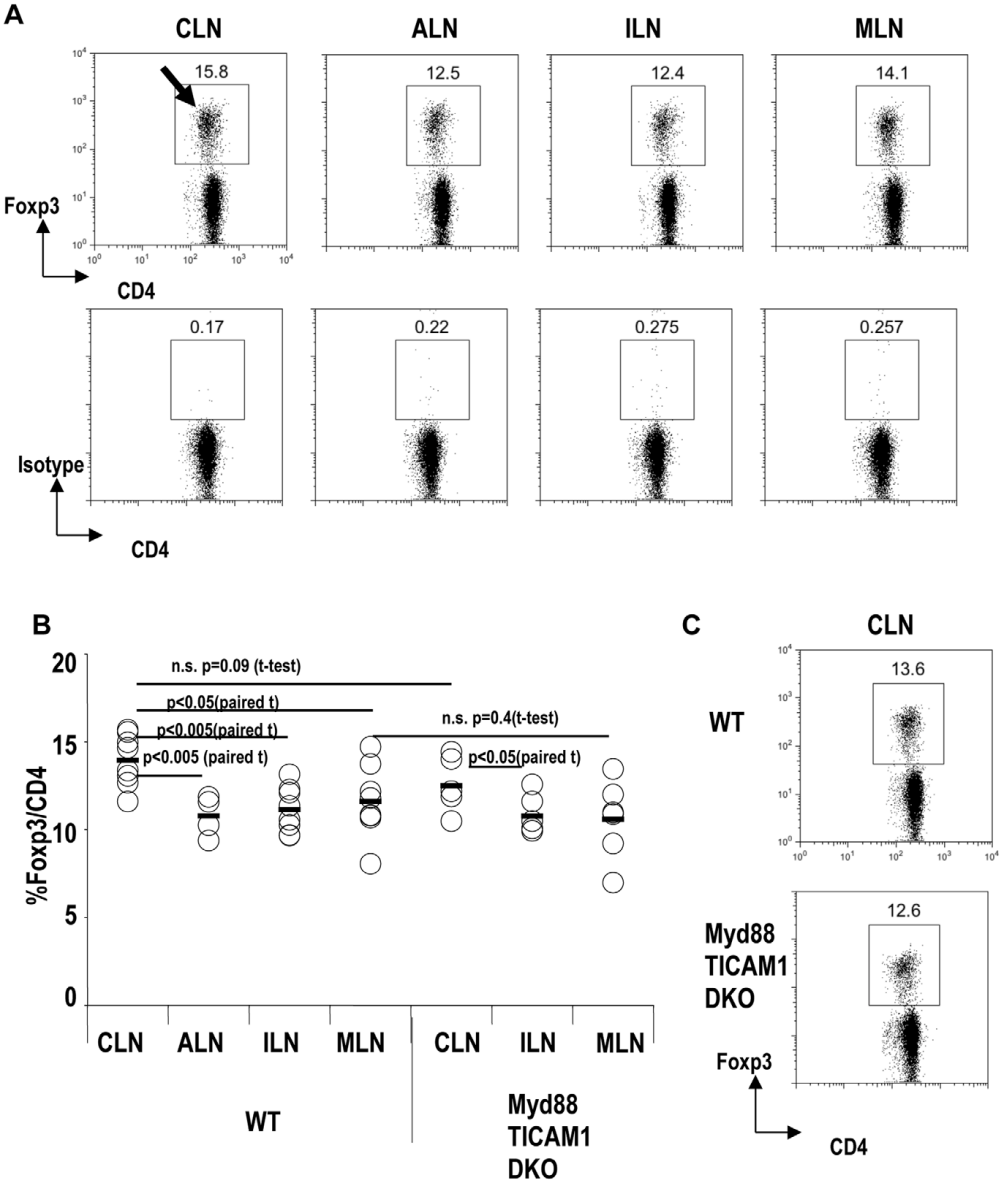
Frequency of Foxp3^+^ T-regs in cervical lymph node is increased in a Myd88/TICAM-1 independent manner. (A) Cervical lymph nodes (CLN), axillary lymph nodes (ALN), inguinal lymph nodes (ILN), and mesenteric lymph nodes (MLN) from wild type B6 mice were analyzed for the expression of Foxp3. The isotype control is shown at the bottom. Plots were gated on CD4^+^ T cells. Representative of seven separate experiments. (B) As in (A), but CLN, ALN, ILN and MLN from wild-type B6 mice (WT) or Myd88/TICAM1 double knockout mice (Myd88/TICAM1 DKO) were analyzed for the expression of Foxp3. Summary from seven separate experiments. P value provided is by paired t-test or t-test. “n.s.” = “not significant”. (C) As in (B), but a representative of CLN is shown.

We considered whether Foxp3^+^T-regs played an important role in the skin or oral cavity because the skin and oral cavity are exposed to many commensal microbes and antigens, like the intestine. First, we investigated the frequencies of Foxp3^+^T-regs in lymph nodes (LNs) at different anatomical locations, which included skin- and oral-cavity-draining LNs. We found that cervical LNs (CLNs) contained a higher frequency of Foxp3^+^ T-regs than other skin-draining LNs, such as axillary LNs (ALNs) and inguinal LNs (ILNs; paired t-test: p<0.005; Fig.1A arrow, Fig.1B and Fig.S1). CLNs contained a slightly, but significantly, higher frequency of Foxp3^+^ T-regs than mesenteric LNs (MLNs; paired t-test p<0.05; Fig.1B). In MLNs, Foxp3^+^T-regs are actively induced by CD103^+^ DCs [16], [17]. These data suggest that Foxp3^+^T-regs may be also induced in CLNs, as in MLNs.

To investigate if the frequency of DCs correlates with the frequency of Foxp3^+^T-regs, the frequency of CD11c^+^DCs in total cells was compared between CLNs and ALNs (Fig.S2). The frequency of DCs was similar between CLNs and ALNs.

Recent reports showed that signals though TLR-2 induce Foxp3^+^T-regs [20], [27], [28]. To examine whether signals from TLRs are required for the high frequency of Foxp3^+^ T-regs in CLNs, we took advantage of Myd88 and TICAM-1 (TRIF) double knockout mice (Myd88/TICAM1 DKO), which lack all TLR signaling [29], [30]. In Myd88/TICAM1 DKO mice, CLNs still contained a significantly higher frequency of Foxp3^+^T-regs than inguinal LNs (ILNs; paired t-test: p<0.05; Fig.1B). The frequency of Foxp3^+^T-regs in CLNs did not differ between Myd88/TICAM1 DKO and wild-type (WT) mice (t-test: p = 0.09; Fig.1B, 1C). The frequency of Foxp3^+^T-regs in MLNs did not differ between Myd88/TICAM1 DKO and WT mice also (t-test: p = 0.4; Fig.1B).

Thus, Foxp3^+^T-regs are increased in CLNs in a Myd88/TICAM1-independent manner, suggesting that TLR signals are not involved in the increase in Foxp3^+^ T-regs in CLNs.

### The Frequency of ROR-γt^+^ CD4^+^T Cells is Increased in CLNs in a Myd88/TICAM1- Dependent Manner

**Figure 2:**
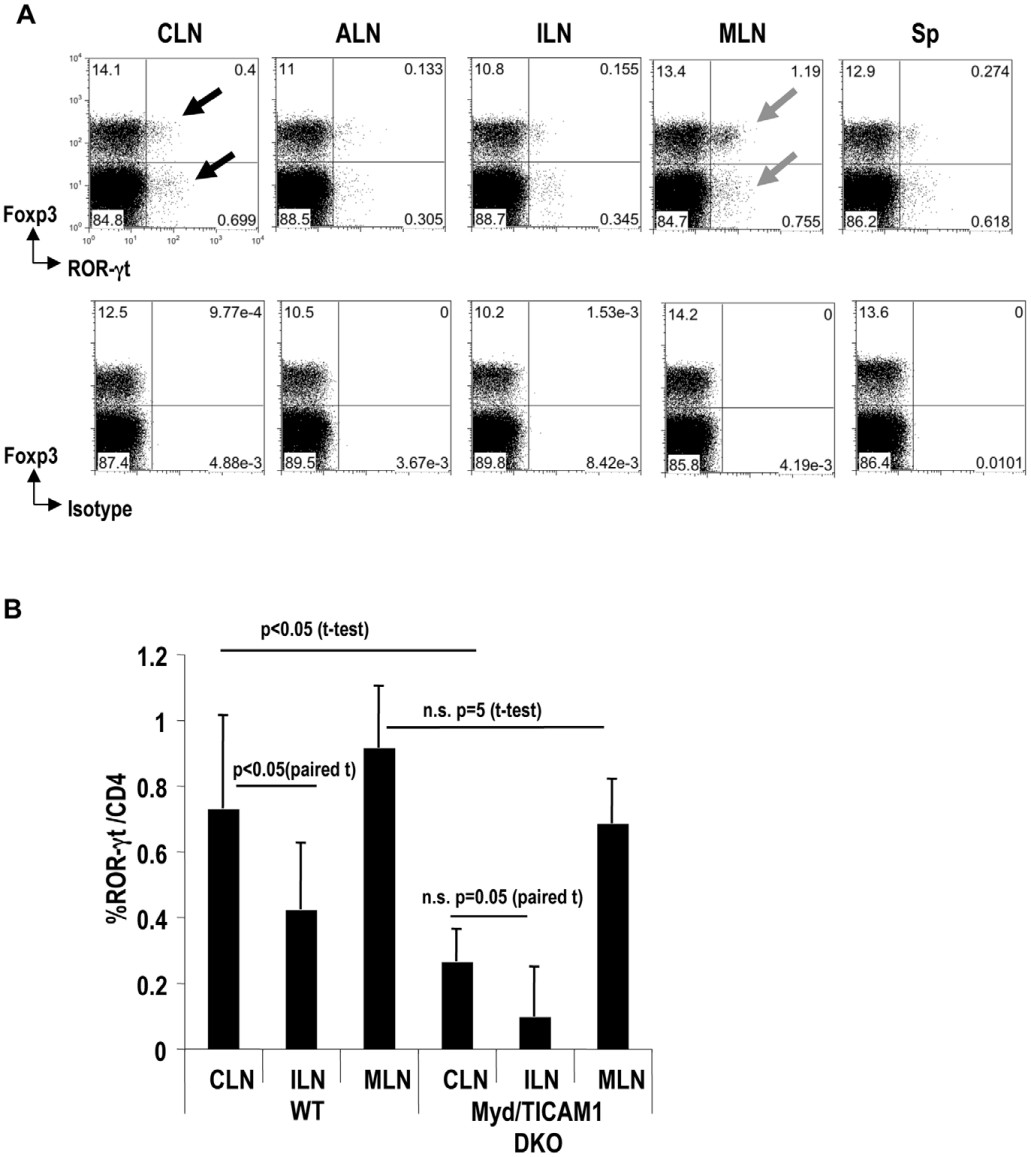
Frequency of ROR-γt^+^CD4^+^ T cell in cervical lymph node is increased in a Myd88/TICAM-1 dependent manner. (A) CLN, ALN, ILN, MLN, and spleen (Sp) from WT B6 mice were analyzed for the expression of Foxp3 and ROR-γt. The plots were gated on CD4^+^ T cells. Isotype staining for ROR-γt is shown at the bottom. Representative of three separate experiments. (B) As in (A), but cells from WT mice or Myd88/TICAM-1 DKO mice were analyzed for the expression of ROR-γt and CD4. The graphic shows a summary from two separate experiments. P value provided is by paired-t test or t-test. “n.s.” = “not significant”.

The induction of Foxp3^+^T-regs in the intestine is reciprocally controlled by Th17 [18], [31]. To examine the balance between Th17 and Foxp3^+^T-regs, we next compared the frequencies of ROR-γt^+^ CD4^+^T cells in different anatomical locations. ROR-γt is a transcription factor expressed by Th17 cells [32]. We found that CLNs had a significantly higher frequency of ROR-γt^+^ CD4^+^T cells than other skin-draining LNs and spleen (paired t-test: p<0.05; Fig.2A closed arrows and Fig.2B). As expected, MLNs contained a higher frequency of ROR-γt^+^ CD4^+^T cells than other LNs (Fig.2A gray arrows and Fig.2B).

To assess whether signals from microbes through TLRs are required for the induction of ROR-γt^+^ CD4^+^T cells in CLNs, we investigated Myd88/TICAM1 DKO mice. In Myd88/TICAM1 DKO mice, frequencies of ROR-γt^+^ CD4^+^T cells did not differ between CLNs and ILNs (paired t-test: p = 0.05; Fig.2B). Moreover, the frequency of ROR-γt^+^ CD4^+^T cells in CLNs was significantly reduced in Myd88/TICAM-1 DKO mice (t-test: p<0.05; Fig.2B). The frequency of ROR-γt^+^ CD4^+^T cells in MLNs did not differ between Myd88/TICAM1 DKO and WT mice also (t-test: p = 0.1; Fig.2B).

Thus, both Foxp3^+^T-regs and Th17 may be induced in CLNs. However, Myd88/TICAM1 signaling is important for the development of ROR-γt^+^CD4^+^-Th17 T cells in CLNs (Fig.2B), but not for the induction of Foxp3^+^T-regs (Fig.1B).

### CLNs are Oral-cavity-draining Lymph Nodes

**Figure 3:**
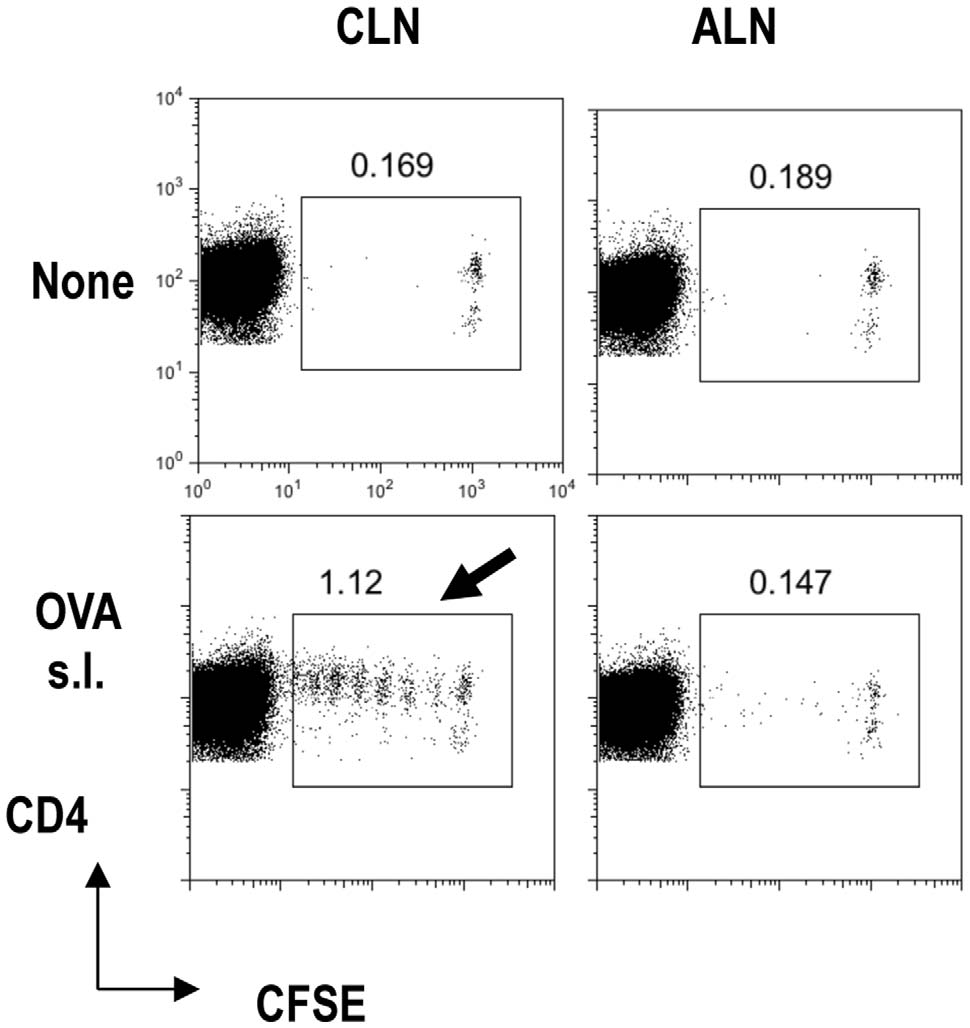
Cervical lymph nodes are draining lymph nodes from the oral cavity. CFSE-labeled OTII CD4^+^T cells were adoptively transferred into B6 mice on day −1. On day 0, 500 µg of OVA was administered sublingually (s.l.). CLN or ALN was analyzed for CFSE dilution at day 3. One of two similar experiments is shown for the FACS plots. Plots were gated on CD4^+^T cells.

We considered that the higher frequency of Foxp3^+^T-regs in CLNs may reflect their response to antigens in the oral cavity. To confirm whether CLNs were draining LNs from the oral cavity, we investigated the proliferation of transferred OT-II CD4^+^-T cells in CLNs after sublingual (s.l.) administration of ovalbumin (OVA; Fig.3). OT-II mice are commonly used OVA-specific CD4^+^ T-cell receptor transgenic mice [7], [10], [28]. In the absence of OVA s.l. administration, carboxyfluorescein diacetate succinimidyl ester (CFSE)-labeled OT-II T cells did not divide, in CLNs or ALNs (Fig.3, top). With OVA s.l. administration, CFSE-labeled OT II T cells divided well in CLNs (Fig.3, bottom arrow), but not in ALNs. Thus, we confirmed that CLNs were draining LNs of the oral cavity because s.l.-administered OVA antigen was presented to OT II CD4^+^-T cells.

### DCs from Oral-cavity-draining CLNs Locate Close to Foxp3^+^T-regs and have the Capacity to Induce Foxp3^+^ T-regs on Antigen Stimulation

**Figure 4:**
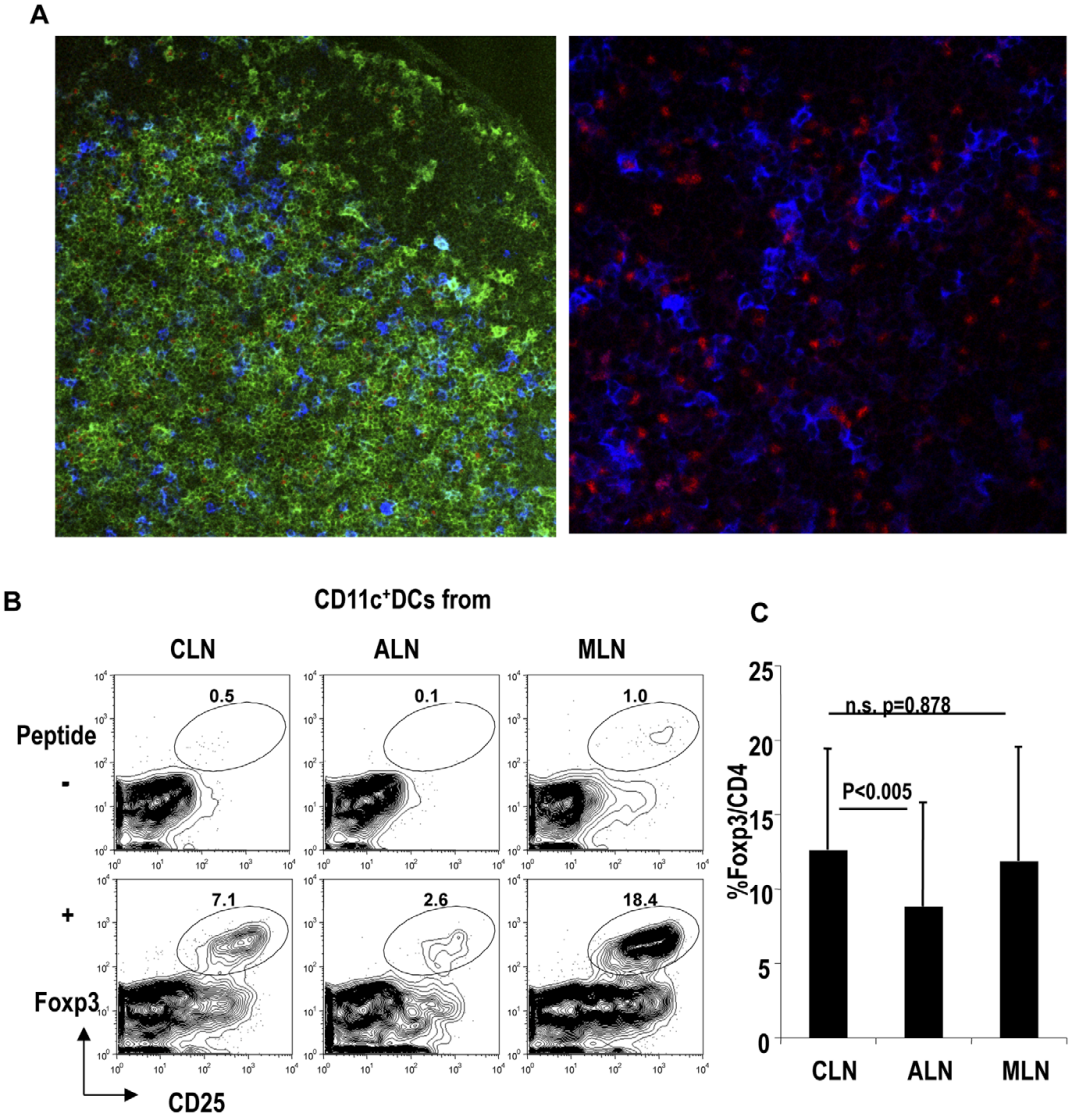
Dendritic cells from oral-cavity-draining cervical lymph nodes induce Foxp3^+^ T-regs. (A) CLNs were stained with Foxp3 (red), CD4 (green) and CD11c (blue). Representative of three similar separate experiments. (B) OT II CD4^+^ T cells (5×10^4^) were cultured with dendritic cells (DCs) from CLN, ALN, or MLN (5×10^4^) with or without OVA peptide. After 5 days, cells were stained with Foxp3, CD25 and CD4. The plots were gated on CD4^+^ T cells. Representative of four separate experiments. (C) As in (A), but the graphic shows a summary of four separate experiments. P value provided is by paired t-test. “n.s.” = “not significant”.

Next, to investigate the interaction between DCs and Foxp3^+^T-regs in CLNs, we microscopically examined CLNs. We found that Foxp3^+^T-regs and CD11c^+^ DCs were closely located, as reported previously in MLNs [33] (Fig.4A). This suggests that DCs from CLNs may induce Foxp3^+^T-regs as DCs do in MLNs.

To determine whether DCs from the oral cavity can in fact induce Foxp3^+^T-regs, we compared the capacity to induce Foxp3^+^T-regs *in vitro* using DCs from ALNs, MLNs, and oral-cavity-draining CLNs. Purified CD11c^+^ DCs from CLNs, ALNs, or MLNs were cultured with OT II CD4^+^T cells with or without antigen for 5 days. In the presence of antigen, CLN DCs induced a higher frequency of Foxp3^+^T-regs compared with ALN DCs (paired t-test: p<0.005; Fig.4B, 4C). The frequency of Foxp3^+^T-regs induced by antigen plus DCs did not differ between the culture with CLN DCs and that with MLN DCs (paired t-test: p = 0.878; Fig.4C).

These results indicated that DCs from the oral-cavity-draining CLNs had the capacity to induce Foxp3^+^T-regs with antigen, as DCs from MLNs do.

### CD103^+^DCs may not be Involved in Inducing Foxp3^+^ T-regs in Oral-cavity-draining CLNs

**Figure 5:**
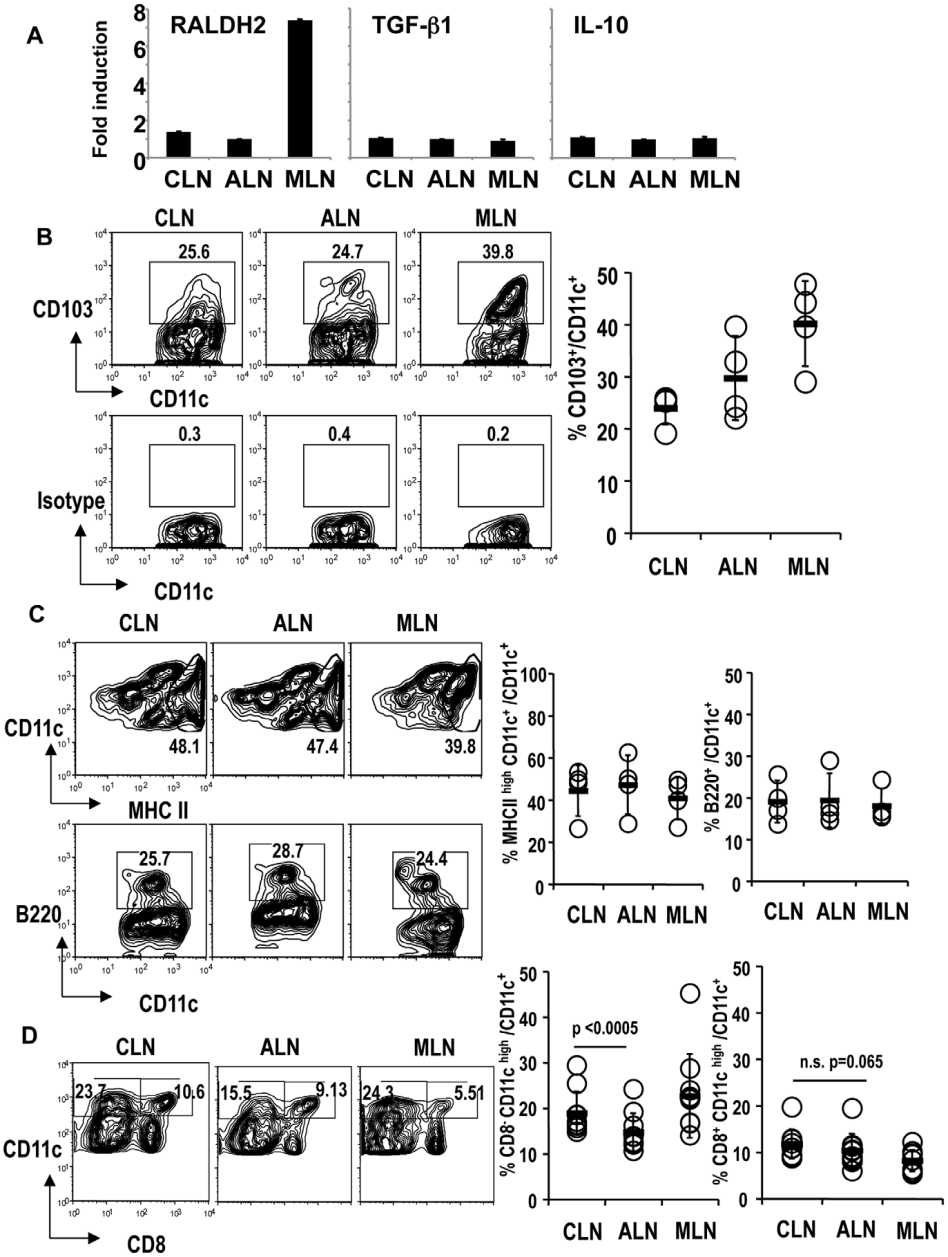
Phenotype of dendritic cells from cervical lymph nodes. (A) DCs from CLN, ALN, and MLN were freshly prepared from B6 mice. mRNA was prepared and real-time PCR was performed. Expression of each sample was normalized to GAPDH mRNA expression and fold increase of each sample was calculated relative to the expression at 0 h. One of two separate experiments is shown. (B) DCs from CLN, ALN, and MLN were analyzed for the expression of CD103. The plots were gated on CD11c^+^ cells. The isotype control for CD103 is shown at the bottom. The graphic shows a summary of four separate experiments. Average +/− SD is shown. (C) As in (B), DCs from CLN, ALN, and MLN were analyzed for the expression of MHC class II or B220. The plots were gated on CD11c^+^ cells. The graphic shows a summary of four separate experiments. Average +/− SD is shown. (D) As in (B), DCs from CLN, ALN, and MLN were analyzed for the expression of CD8. The plots were gated on CD11c^+^ cells. The graphic shows a summary of 10 separate experiments. P value provided is by paired t-test. “n.s.” = “not significant”.

To determine whether DCs from the oral cavity contain a specific DC subset to induce Foxp3^+^T-regs as in the intestine, we first performed real-time PCR. When we investigated the mRNA expression of retinal dehydrogenase 2 (RALDH2), transforming growth factor (TGF)-ß, and IL-10, there was no difference between DCs from CLNs and ALNs (Fig.4A). DCs from MLNs had higher mRNA expression of RALDH2 as previously reported (Fig.5A). We also measured the protein production of TGF-ß1 and IL-10 in the culture supernatant. TGF-ß1 was not detected in the culture supernatants of CLN DCs with or without latent TGF-ß activation (data not shown). We did not detect IL-10 in the culture supernatants from CLN DCs and OT II CD4^+^T cells without peptide in Fig.4B and 4C (data not shown). Theses results indicate that TGF-ß1, IL-10 and RALDH2 may not involve in the induction of Foxp3^+^T-regs by CLN DCs.

To investigate whether CD103^+^DCs play a role in inducing Foxp3^+^T-regs in CLNs, we compared the frequency of CD103^+^DCs in each location. However, oral cavity-draining CLNs had a lower frequency of CD103^+^ DCs than MLNs (Fig.5B).

Plasmacytoid DCs have a capacity to induce Foxp3^+^T-regs [34], [35], [36], [37]. Epidermal Langerhans cells and migratory dermal DCs have also been reported to induce Foxp3^+^T-regs [38], [39], [40]. However, the frequencies of plasmacytoid DCs and migratory class II^high^ DCs did not differ between CLNs and ALNs (Fig.5C). Next, we investigated the classical CD8^+^ and CD8^−^ DC subsets in CLNs and ALNs. The frequency of CD8^+^ DCs was similar between CLNs and ALNs (paired t test: p = 0.065) (Fig.5D). However, CLNs had a significantly higher frequency of CD8^−^ DCs than ALNs (paired t test: p<0.0005) (Fig.5D).

These results suggest that CD103^+^ DCs and retinoic acid may not contribute to inducing Foxp3^+^T-regs in CLNs. It is possible that the classical CD8^−^ DC subset in CLNs may participate in the induction of Foxp3^+^T-regs. Further studies are required.

## Discussion

The oral cavity is exposed to many antigens and commensal organisms every day [23], [24], [25], [26]. The oral cavity is frequently associated with systemic immunological disorders, such as graft versus host diseases, Stevens-Johnson syndrome and Behçet diseases. However, it is unknown how tolerance in the oral cavity is maintained. Here, we showed that the balance between Th17 and Foxp3^+^T-regs may play a role in maintaining tolerance in the oral cavity. We found that the frequencies of Foxp3^+^T-regs and ROR-γt^ +^CD4^+^T cells were increased in oral-cavity-draining CLNs, compared with skin-draining LNs and mesenteric LNs. DCs from oral-cavity-draining CLNs have the capacity to induce Foxp3^+^ T-regs *in vitro* on antigen stimulation, as much as DCs from mesenteric LNs do. These data suggest that the induced Foxp3^+^T-regs in oral-cavity-draining CLNs may be important in maintaining mucosal tolerance in response to microbes and food antigens in the oral cavity.

Although some TLR signaling is involved in inducing Foxp3^+^ T-regs [20], [27], [28], [41], [42], the high frequency of Foxp3^+^ T-regs in CLNs was not dependent on Myd88/TICAM1 (Fig. 1). Thus, TLR signaling is apparently not involved in the induction of Foxp3^+^T-regs in the oral cavity. However, we cannot exclude the possibility that some specific microbes may be involved in the inducing Foxp3^+^T-regs in the oral cavity in a Myd88/TICAM1 independent manner. For example, Atarashi et al recently showed that *Clostridum* induces Foxp3^+^T-regs in the colon in a Myd88-independent manner [19]. It might be interesting to investigate if there are specific microbes that contribute to the induction of Foxp3^+^T-regs in the oral cavity, especially as the bacterial community varies between the oral cavity and gut [26].

Our results showed that the high frequency of ROR-γt^+^CD4^+^T cells was dependent on Myd88/TICAM1 in the oral-cavity-draining CLNs (Fig.2). This is consistent with recent findings in skin [43]. Th17 cells in skin are reduced in Myd88/TICAM1 knockout mice and skin-resident commensal bacteria induce Th17 cells in a Myd88- and IL-1 receptor-dependent manner [43]. It is also known that some microbes induce Th17 using TLR signals; for example, Th17 cells induced by *Chlamydia* infection are reduced in Myd88 KO mice [44]. Thus, it is possible that TLR signals through some oral microbes are responsible for the increase of ROR-γt^ +^CD4^+^T cells in oral-cavity-draining CLNs. Interestingly, the intestine may use a different mechanism from the oral cavity and skin to maintain Th17 cells, because Th17 cells are not reduced in the intestine in Myd88/TICAM1(TRIF)-knock out mice [45], [46].

Furthermore, we found that DCs from oral-cavity-draining CLNs induce Foxp3^+^T-regs in the presence of antigen, as do DCs from MLNs (Fig.4B). It has been reported that cutaneous CD103^+^DCs induce Foxp3^+^T-regs using RALDH2, as intestinal CD103^+^DCs do [47]. Here, we would like to propose that DCs in the oral cavity use a different mechanism(s) to induce Foxp3^+^Tregs from DCs in the intestine. First, CLNs have few CD103^+^ DCs compared with MLNs (Fig. 5B). Second, DCs from CLNs do not express RALDH2 at the mRNA level (Fig. 5A). We have not yet found any specific DC subset in the oral-cavity-draining CLNs. However, CD8^−^ classical DCs are increased in CLNs versus ALNs. Our previous report showed that CD8^+^DEC205^+^DCs induce Foxp3^+^T-regs from Foxp3^−^ cells and that CD8^−^33D1^+^DCs expand natural occurring Foxp3^+^T-regs [6], [11]. Thus, it seems possible that CD8^−^DCs in CLNs may participate in expanding natural occurring Foxp3^+^T-regs.

Recently, it has been reported that recently activated Foxp3^+^T-regs from CLNs accumulated in CLNs after adoptive transfer [48]. It was suggested that TCR-mediated signals upon antigen stimulation may play a key role in the site-specific accumulation of Foxp3^+^T-regs in CLNs. Taken together, oral-cavity-draining CLNs may be a special location where Foxp3^+^T-regs are induced and also accumulate.

Here we showed that Foxp3^+^T-regs are induced in oral-cavity-draining CLNs in a Myd88/TICAM1 independent manner and that DCs from oral-cavity-draining CLNs have the capacity to induce Foxp3^+^Tregs on antigen stimulation. The mechanisms by which DCs to induce Foxp3^+^T-regs may differ from those in the intestine. We propose that Foxp3^+^T-regs play an important role in maintaining tolerance in the oral cavity to suppress Th17, as in the intestine. DCs from CLNs play a key role in maintaining tolerance upon oral antigen stimulation in the oral cavity. Further studies are required to identify the mechanism(s) by which DCs to induce Foxp3^+^T-regs in the oral cavity.

## Materials and Methods

### Mice

C57BL6J (B6) mice were from Japan Clea (Tokyo). Myd88 KO mice were from Dr. Shizuo Akira (Osaka University). TICAM-1 KO mice were established in our laboratory [29], [30]. OT-II OVA CD4 T cell receptor transgenic mice were kindly provided by Dr. Kazuya Iwabuchi (Kitasato University). The mice were maintained in the Hokkaido University Animal Facility in a specific pathogen-free condition. All experiments used mice between 6-12-week-old mice at the time of first procedure. All mice were used according to the guidelines of the institutional animal care and use committee of the Hokkaido University, who approved this study (ID number: 08-0243, “Analysis of immune modulation by toll-like receptors.”).

### Antibodies and Reagents

PE-conjugated CD103, CD25 (PC61), Alexa-488 conjugated anti-CD25 (7D4), FITC, biotin or APC conjugated CD4 (RM4-5), CD11c, B220, NK1.1, purified anti-CD16/CD32 (2.4G2) antibodies were from Biolegend (San Diego, CA). Anti-CD11c, and streptavidin microbeads were from Miltenyi Biotec (Gladbach, Germany). CFSE was from Molecular Probes (Eugene, OR). PE conjugated anti-mouse ROR-γt antibody and the anti-mouse Foxp3 (FJK-16s) staining kit were from eBioscience (San Diego, CA). LPS free OVA protein was from Seikagaku Co.(Tokyo, Japan).

### Cell Isolation

CD4^+^ T cells were first negatively separated by MACS beads from lymph nodes and spleen cell suspensions (>90%; Miltenyi Biotech). CD4^+^ T cells were sometimes further purified by FACS Aria II (BD Bioscience, Franklin Lakes, NJ). CD11c^+^ DCs from spleen, CLNs, ALNs, or MLNs were selected with anti-CD11c beads (Miltenyi Biotech) [7], [10].

### Co-culture with T cells and DCs

CD4^+^T cells from OT II transgenic mice were cultured with DCs at 0 or 0.01-µM OVA peptide for 5 days. After 5 days, each culture was stained with Foxp3, following the manufacturer’s protocol. Cells were acquired by FACS calibur flow cytometer (BD). Analyses were performed using the Flowjo software (TreeStar, USA).

### Adoptive Transfer of OT-II CD4^+^T cells

CD4^+^T cells from OT II transgenic mice were labeled with 5 µM CFSE, and 1×10^6^ T cells were injected intravenously into B6 recipients. One day later, OVA protein was administered sublingually. After 3 days, mice were sacrificed, and CLNs and ALNs were stained with CD4 and CFSE dilution was investigated. Cells were assessed by FACS calibur (BD). Analyses were performed using the Flowjo software (TreeStar, USA).

### Quantitative PCR

Total RNA was isolated with TRIzol (Invitrogen), and reversed-transcribed with the High Capacity cDNA Transcription Kit (ABI) according to manufacturer’s instructions. qPCR was performed with the Step One Real-Time PCR system (ABI). All primers for real-time PCR have been reported previously [27], .

### Measuring Cytokine Production

The purified DCs (1×10^5^) were cultured in serum free RPMI medium for 20 h. The concentrations of TGF-ß in the supernatants were measured by TGF-ßELISA kit (R&D). Following the manufacturer’s instructions, we measured the TGF-ß with or without activation of the latent form of TGF-ß. Culture supernatants with OT II CD4^+^T cells and DCs were measured for IL-10 by ELISA (eBiosciences) or Cytometric Bead Array (BD Bioscience). Analysis with the Cytometric Bead Array was performed according to the manufacturer’s instructions.

### Confocal Microscopy

CLNs were sectioned, fixed with acetone, and stained with anti-CD4-FITC and CD11c-APC antibodies. After permeabilization with the buffer from the Foxp3 staining kit (eBioscience), they were stained with an anti-Foxp3-PE antibody. They were washed and observed by confocal microscopy (LSM510 META, Zeiss, Jena, Germany).

## Supporting Information

Figure S1 **CD25^+^ and CD25^−^ Foxp3^+^ T-regs in lymph nodes and spleen.** (A) CLN, ALN, ILN, MLN and Sp from B6 mice were analyzed for the expression of Foxp3 and CD25. The isotype control for Foxp3 is shown at the bottom. Plots were gated on CD4^+^ T cells. Representative of 2 separate experiments is shown. (B) As in (A), but the frequency of CD25^+^ or CD25^−^ Foxp3^+^ T-regs/CD4+T cells were shown.
(TIF)

Figure S2 **The frequency of CD11c^+^ DC is similar between CLN and ALN.** (A) CLN or ALN from one B6 mouse were digested by collagenase and stained with anti-CD11c and CD8 Abs. Representative of 5 separate experiments is shown. (B) The frequency of CD11c^+^ cells/total LN cells in one mouse is shown. A summary of 5 separate experiments. P value provided is by paired t-test. “n.s.” = “not significant”.
(TIF)
