# Supplementary figures and images for: Dendritic Cells from Oral Cavity Induce Foxp3+ Regulatory T Cells upon Antigen Stimulation
Source: PLoS One. 2012 Dec 18;7(12):e51665. doi: 10.1371/journal.pone.0051665 (PMC3525649; doi:10.1371/journal.pone.0051665)

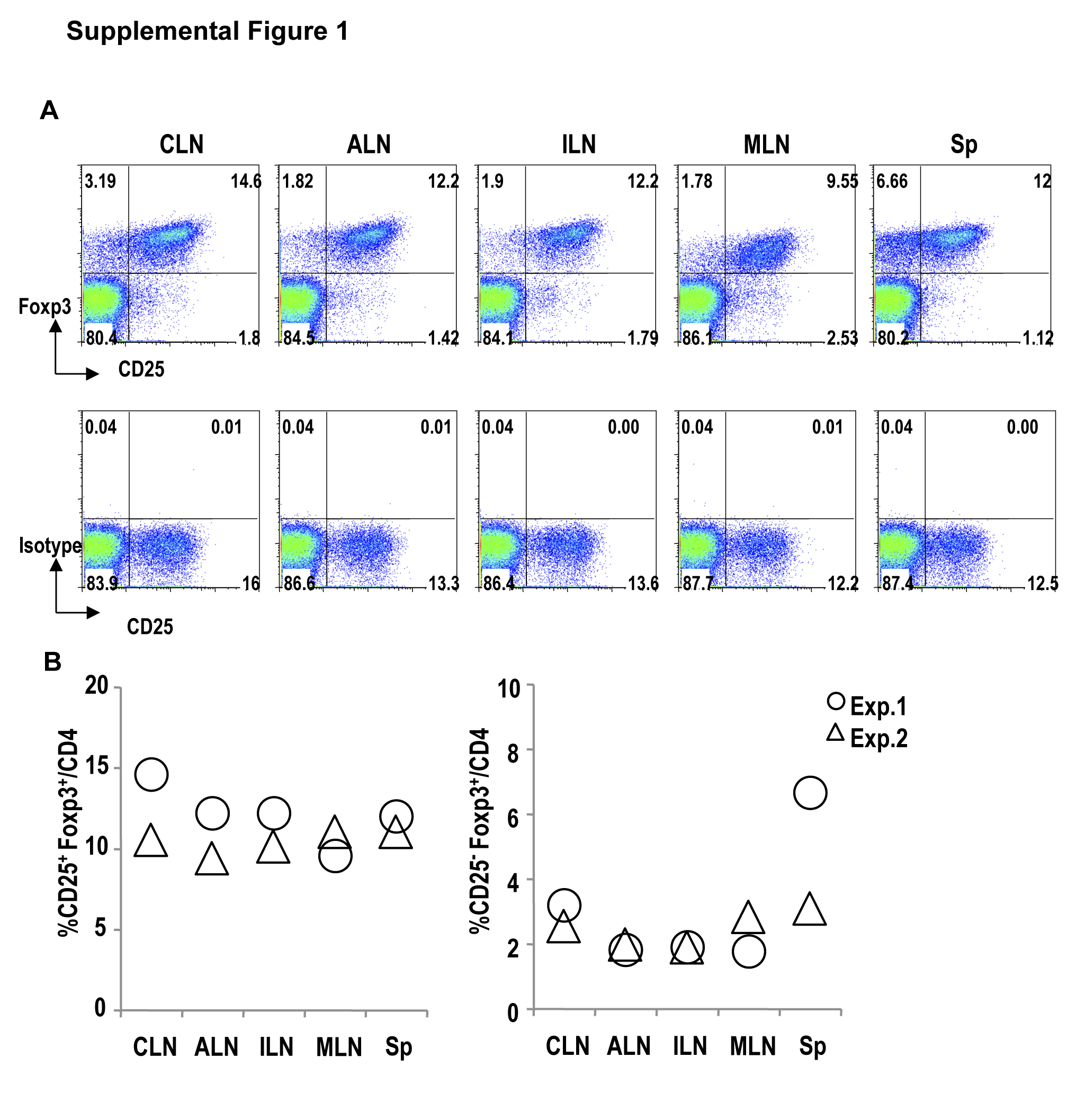

Supplement: Figure S1 — CD25+ and CD25− Foxp3+ T-regs in lymph nodes and spleen. (A) CLN, ALN, ILN, MLN and Sp from B6 mice were analyzed for the expression of Foxp3 and CD25. The isotype control for Foxp3 is shown at the bottom. Plots were gated on CD4+ T cells. Representative of 2 separate experiments is shown. (B) As in (A), but the frequency of CD25+ or CD25− Foxp3+ T-regs/CD4+T cells were shown. (TIF) [file pone.0051665.s001.tif]

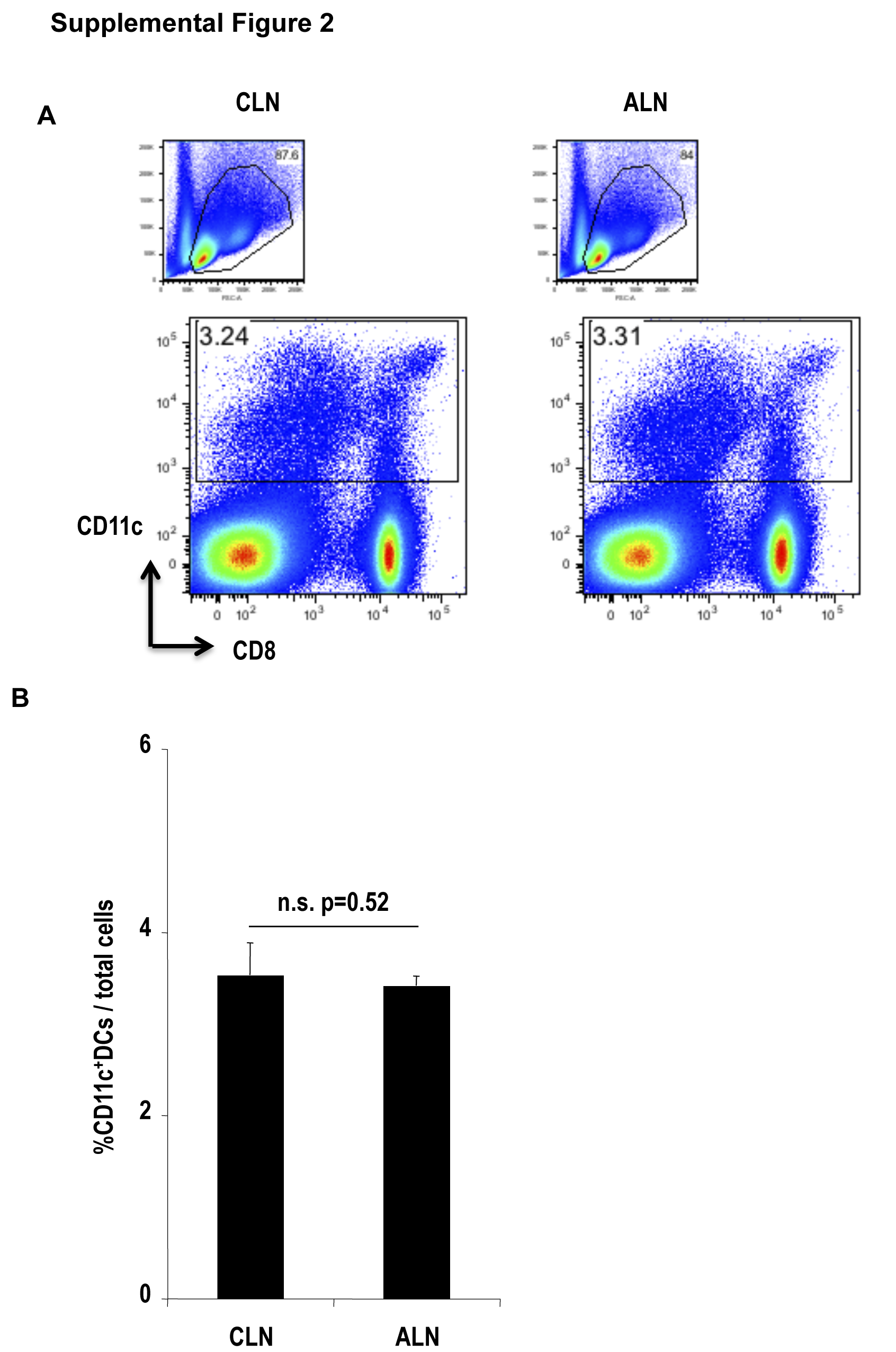

Supplement: Figure S2 — The frequency of CD11c+ DC is similar between CLN and ALN. (A) CLN or ALN from one B6 mouse were digested by collagenase and stained with anti-CD11c and CD8 Abs. Representative of 5 separate experiments is shown. (B) The frequency of CD11c+ cells/total LN cells in one mouse is shown. A summary of 5 separate experiments. P value provided is by paired t-test. “n.s.” = “not significant”. (TIF) [file pone.0051665.s002.tif]
